# Desorption from Hot Scandate Cathodes: Effects on Vacuum Device Interior Surfaces after Long-Term Operation

**DOI:** 10.3390/ma13225149

**Published:** 2020-11-16

**Authors:** Mujan N. Seif, T. John Balk, Matthew J. Beck

**Affiliations:** Department of Chemical and Materials Engineering, University of Kentucky, Lexington, KY 40506-0046, USA; mujan.seif@uky.edu (M.N.S.); john.balk@uky.edu (T.J.B.)

**Keywords:** scandate cathodes, desorption, vacuum electronics, electron devices

## Abstract

Scandate cathodes have exhibited superior emission properties compared to current state-of-the-art “M-type” thermionic cathodes. However, their integration into vacuum devices is limited in part by a lack of knowledge regarding their functional lifespan and behavior during operation. Here, we consider thermal desorption from scandate cathodes by examining the distribution of material deposited on interior surfaces of a sealed vacuum device after ~26,000 h of cathode operation. XPS, EDS, and TEM analyses indicate that on the order of 1 wt.% of the initial impregnate is desorbed during a cathode’s lifetime, Ca does not desorb uniformly with time, and little to no Sc desorbs from the cathode surfaces (or does so at an undetectable rate). Findings from this first-ever study of a scandate cathode after extremely long-time operation yield insight into the utility of scandate cathodes as components in vacuum devices and suggest possible effects on device performance due to deposition of desorption products on interior device surfaces.

## 1. Introduction

Thermionic cathodes are electron sources used in a wide range of vacuum electron devices (VEDs) critical to both military and civilian applications. Examples include devices based on RF amplifying traveling wave tubes—satellite communication systems and military/civilian RADAR applications [1]), miniature thermionic diodes [2], and more [3,4]. To meet increasing demand, high-power cathodes are being sought for THz or mm-wave devices [5] operating beyond the crowded kHz, MHz, and GHz frequency ranges. Delivering the necessary enhanced current densities while lowering operating temperatures requires improvements to existing cathode designs or the development of next-generation electron emitting materials.

A significant refinement of uncoated thermionic dispenser cathodes [6,7] has been reported with the addition of Sc or Sc${}_{2}$O${}_{3}$ to the powder-processed porous W matrix [8]. While Sc-containing (“scandate”) cathodes have exhibited higher emitted current densities than other dispenser cathodes (100–400 A/cm${}^{2}$ at ~850 ${}^{\circ }$C [9,10]), inconsistent performance and poor reproducibility have prevented scandate cathodes from being widely integrated into devices [11]. In addition, little is known about the amount of and manner by which the Ba-Ca-Al impregnate desorbs from scandate cathodes and the location within a vacuum device these materials may redeposit.

In the following, measurements of the distribution of re-deposited desorption products after activation and operation of a set of scandate cathodes are reported. Particular attention is given to the fate of both Ca and Sc, as the addition of these elements to the cathode is known to improve electron emission [6,7,9,10]. The desorption of Ca is of particular interest because recent compositional analysis of cathodes impregnated with a CaO-containing oxide mix did not locate Ca in the cathodes post-activation [12]. To assess the fate of chemical species desorbed from cathode surfaces, X-ray Photoelectron Spectroscopy (XPS) and energy dispersive X-ray spectroscopy (EDS) studies were conducted on components of a sealed glass envelope in which scandate cathodes had been activated and operated for ~26,000 h. Emission testing, while not the focus of this study, has been presented by the manufacturer for similar cathodes elsewhere [13,14]. Combined with information about the activation and operating conditions after the vacuum envelope was sealed, the present results show that cathode activation and operation occur in an oxygen-poor environment, that little to no Sc is desorbing from the cathode surface, and that, while impregnate material is deposited on the anode itself, this material accounts for only on the order of 1% of all impregnate added during fabrication. These findings from this first-ever study of scandate cathodes after long-term operation provide new insight into the fitness of scandate cathodes for use as critical components in vacuum devices and suggest potential impacts on device performance due to deposition of desorption products on interior device surfaces.

## 2. Methods and Materials

### 2.1. Device Elements

The cathodes investigated in this work were fabricated and life-tested by collaborators at eBeam, Inc. (Beaverton, OR, USA). The cathode fabrication process is largely proprietary; however, certain general practices are known. The cathodes are comprised of a porous W matrix doped with ~4 wt.% Sc${}_{2}$O${}_{3}$ by the liquid–liquid (L-L) method [15], then die pressed into a loose pellet. A near-eutectic mixture of solid BaCO${}_{3}$-CaCO${}_{3}$-Al${}_{2}$O${}_{3}$ with a molar ratio of 6:1:2 was calcined in air until it reduced to a BaO-CaO-Al${}_{2}$O${}_{3}$ powder. The oxide impregnate powder was distributed across the top of the cathode pellet, which was then heated above 1500 ${}^{\circ }$C, and the (now liquid) oxide mixture infiltrated the pellet in a process called “impregnation”. Excess impregnate on the macroscopic cathode surface was removed mechanically and the cathode surface was then washed with deionized water. The cathodes were then placed on individual W/Re wire heaters encompassed in Mo/Re sleeves.

**Figure 1:**
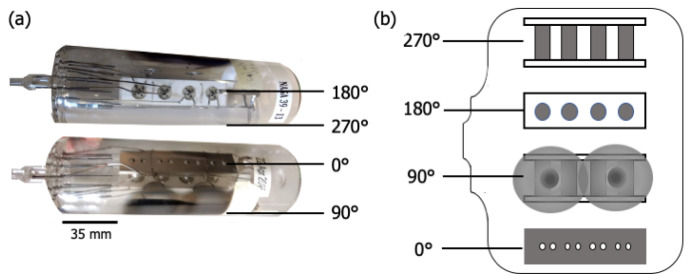
(**a**) Photographs of the scandate cathode test vehicle, comprised of a glass envelope containing the cathode assembly, and (**b**) an “unrolled” schematic highlighting different views around the circumference of the envelope.

**Figure 2:**
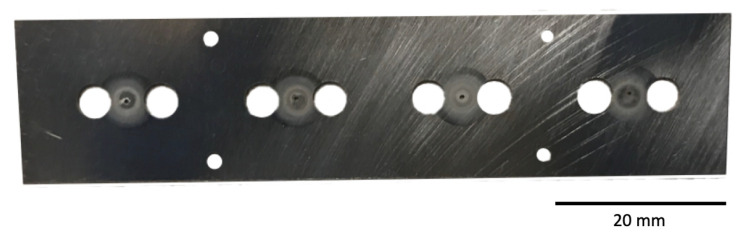
A photograph of the side of the anode facing the cathode components. During testing, the small pinholes at the center of each discolored ring are directly in line with the cathodes.

Figure 1 shows two images of (a) the glass envelope containing the cathode assembly, and (b) an “unrolled” schematic highlighting views at right angles around the circumference of the envelope. Figure 2 shows the anode plate after removal from the envelope and cathode assembly. Once the cathode assembly was inserted, the glass envelope was mechanically pumped, and the cathodes were heated to ~1000 ${}^{\circ }$C${}_{b}$ (brightness temperature) for ~1 h. This initial heating stage is referred to as “pre-activation”. The envelope was then sealed by heating and pinching closed a molten glass nipple around the leads (left end of envelope in Figure 1). To remove remaining gases trapped within the envelope, pans of metallic Ba-Al alloy included as part of the cathode assembly were flashed to a high temperature, sublimating Ba and Al, and gettering residual gases to yield a final vacuum atmosphere. Residue from the two flash pans is apparent along the 90${}^{\circ }$ longitude in Figure 1. The enclosed cathodes were then “activated” by heating with their attached heaters to ~1150 ${}^{\circ }$C${}_{b}$ for 1–2 days. The cathodes assembled in the test vehicle studied here were then operated for ~26,000 h at ~950 ${}^{\circ }$C${}_{b}$ and exhibited an emission of ~1–5 A/cm${}^{2}$.

After testing, the entire sealed test vehicle (including the cathodes) was sent to the University of Kentucky for opening, disassembly, and analysis. The test vehicle arrived to the authors in the same configuration and condition it was in after testing conducted at eBeam, Inc. To explore the fate of species desorbed from the cathodes during pre-activation, activation, and/or operation, two elements of the cathode test vehicle were examined: (1) the interior surface of the glass envelope enclosing the cathode assembly and (2) the Mo anode positioned in the cathodes’ line-of-sight.

### 2.2. Sample Preparation

Before disassembly, the exterior of the envelope was colored with permanent marker according to areas of interest: directly opposite the assembly surface (0${}^{\circ }$ in Figure 1), directly opposite the assembly base (180${}^{\circ }$), the Ba-Al flash region (90${}^{\circ }$), and diametrically opposite the Ba-Al flash (270${}^{\circ }$). Two controls were also marked: the end of the cathode opposite the nipple, which served as a control on the interior of the envelope, and another portion of this same area, which was a control on the exterior of the envelope, used to attain the baseline composition of the glass. The coloring was done to ensure that, once broken, the area of origin of each shard of the envelope would be known, as well as which side of each shard was the interior. Each area of interest was covered in transparent tape to keep shards in place, and the perimeter of each area was scored with a sapphire scribe to guide fracture.

The glass envelope was broken with a precision hammer in an open plastic container in air in a vented hood. Once broken, samples of the envelope were cut along the previously applied scores between sections. The cathode assembly was removed from the remains of the glass envelope and the anode plate was removed from the assembly. The anode was cut in half with shears, and the two halves stored with opposites sides facing up to preserve sample conditions. Fragments from the glass envelope were placed in separate containers based on region of origin, and every effort was made not to disturb what had been interior surfaces.

### 2.3. Surface Compositional Analysis

A K-Alpha X-ray Photoelectron Spectroscopy system (Thermo Fisher Scientific, Hillsboro, OR, USA) was used to analyze surface composition. Both interior and exterior surfaces of the envelope were studied, and only the external surface of the glass exterior control sample was cleaned in any fashion. The glass envelope and anode were studied in two separate but similar XPS experiments. The samples were secured to the sample stage with copper pins. An Al Kα micro-focused monochromator with a spot size of 400 μm was used, and provided a probe sampling depth of ~10 nm. An electron flood gun was used to maintain charge neutrality of insulating samples. For each point studied on the glass samples, a full spectrum survey composed of 20 individual survey scans as well as targeted surveys for Ba, Ca, Sc, Al, W, Si, C, and O were recorded. Measurement conditions were the same for anode samples, except that the flood gun was not used and a targeted scan for Mo was substituted for Si.

Calibration of XPS results for sample regions inside the glass envelope was accomplished by shifting the binding energies so that the Ba 3d peaks matched known positions of 780.0 eV and 795.3 eV. The latter peak (present due to spin-orbit effects) directly overlaps with Co2p peaks; however, the ratio of the primary 3d peak and the spin-orbit peak is 3:2 for Ba, which was observed for the present samples [16]. Traditionally, binding energies obtained via XPS are calibrated by shifting to align the adventitious carbon peak. However, as a range of carbonates (which complicate identification of the adventitious C peak) may be present inside the envelope and Ba is present in all tested interior samples, energies were shifted according to Ba. Results from the envelope’s exterior, obtained to determine baseline glass composition, were calibrated with the adventitious C peak.

Quantitative analysis of peak heights is presented in the form of signal-to-noise (S/N) ratios to allow comparison between scans. Noise levels were calculated as the standard deviation of measured counts in a binding energy range without peaks for each spectrum. Typical noise levels were ~2000 measured counts with maximum peak intensities typically two orders of magnitude greater than the noise. Background-corrected counts for each peak were divided by the noise level to give S/N ratios in multiples of noise level. In subsequent discussion, peak intensity is considered to be “weak” or “trace” if the peak height as a multiple of the computed noise level (S/N ratio) is less than 3, “moderate” if it is in a range of 4 to 15, and “strong” if it exceeds 16.

Additional cross-sectional transmission electron microscope (TEM) analysis was conducted on a glass sample taken from the Ba-Al flash region and an anode sample taken from a heavily deposited region opposite a cathode emitting surface. The two corresponding lift-out samples were fabricated with a dual-beam focused-ion beam (FIB) and imaged with scanning electron microscopy (SEM), utilizing an FEI Helios NanoLab 660 (Thermo Fisher Scientific, Hillsboro, OR, USA). Elemental mapping of the lift-out samples was conducted with an FEI Talos F200X TEM (Thermo Fisher Scientific, Hillsboro, OR, USA) equipped with a Super-X EDS system (which is comprised of four confocal detectors that simultaneously collect X-rays from the same location). The whole anode deposit area was characterized with SEM and elemental mapping obtained with the Oxford X-Max EDS 80 mm${}^{2}$ detector (Oxford Instruments, Abingdon, UK)-equipped FEI Helios NanoLab 660.

## 3. Results & Discussion

### 3.1. Analysis of Glass Envelope

**Figure 3:**
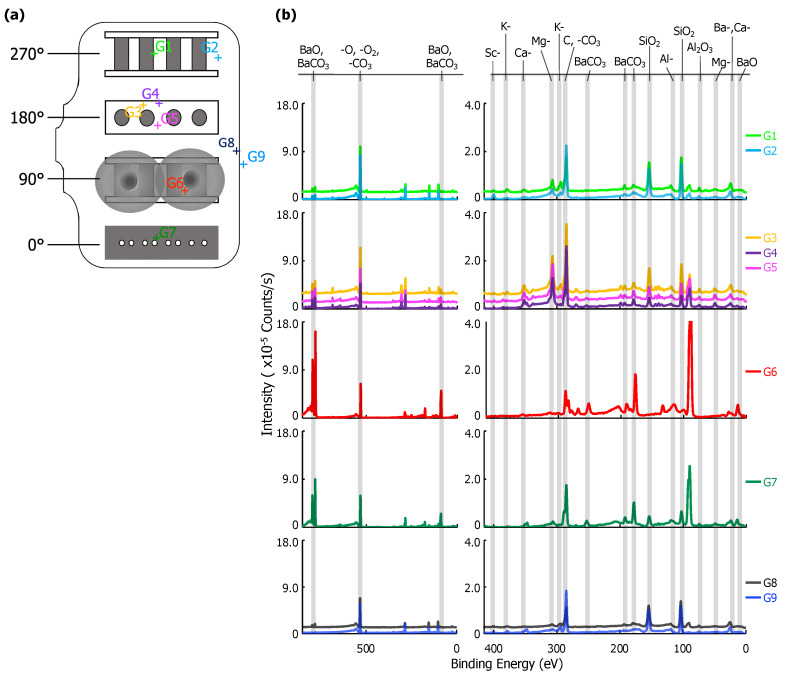
(**a**) A diagram of the glass envelope introduced in Figure 1 plotted with each examined point, G1–G9. (**b**) On the left, the full X-ray Photoelectron Spectroscopy (XPS) survey of each point scan with the three highest intensity peaks identified. Note that there is an arbitrary vertical shift applied to G1, G4, G5, and G8 to improve readability. On the right, a plot of peaks corresponding to binding energies from 400 to 0 eV. Labels for each relevant peak are included.

XPS point scans from eight positions on the interior and one position on the exterior of the glass envelope are shown in Figure 3. Arbitrary vertical shifts were applied to facilitate plotting multiple spectra. Full scans (850–0 eV) are generally dominated by the Ba-, O-, and C- peaks above 400 eV. Peaks below 400 eV are highlighted in a separate spectra; both are included in Figure 3b.

The location of each point scan is indicated in Figure 3a. G7 is above the cathodes opposite the anode plate, which has pinholes with ~0.5 mm diameters directly in line with the cathodes (see Figure 1 and Figure 2). G1 and G2 are adjacent to the cathode assembly, and G6 is within the Ba-Al flash region. G3, G4, and G5 are behind the cathodes (see Figure 1). G8 is a reference interior point opposite the sealed opening, and G9 is a similarly located exterior reference. Peak binding energies, background-deducted counts, S/N ratios, spectral lines, and species identification for each position can be found in tables located in the Appendix A.

Six elements were observed in XPS scans at various points on the glass envelope: Al, Ba, Ca, K, Mg, and Si. Ba signal, likely originating from BaO and BaCO${}_{3}$, was observed on all interior surfaces, but were absent from the exterior reference point (G9). The strongest Ba signal was observed at G6, which is directly opposite the Ba-Al flash pans, while trace Ba signal was observed at G8, an interior point far from the flash pans and the cathodes. Moderate SiO${}_{2}$ signal was observed everywhere except at point G6 (Ba-Al flash region), and its strength decreased noticeably at G7 (interior point in front of cathodes). Trace K, Mg, and Ca signal appeared everywhere SiO${}_{2}$ is present; however, K is absent at G7 and Mg signal is significantly enhanced at G1-G5. Al signal was observed at all points. Signal plausibly originating from Sc was only observed at G2, where its S/N ratio = 4. However, it is important to note that the Mo 3p3/2 peak overlaps that of Sc 2p3/2. As Mo is the primary component of the anode, it cannot be asserted with absolute certainty that it is absent on the glass.

Results from G9 give a baseline for the glass envelope itself: Al, Ca, K, Mg, and Si are all present. These results indicate a standard aluminosilicate glass with K, Ca, and Mg dopants [17]. Ca and Al are also both present in the impregnate (known from processing history), but K, Mg, and Si are unique to the glass. Therefore, the absence of K, Mg, and Si at G6 shows that the layer deposited by the Ba-Al flash in this region is thicker than 10 nm, the sampling depth for XPS.

The spectrum at G8 differs from that of G9 only by the presence of moderate Ba signal, showing that Ba from the Ba-Al flash and/or impregnate desorption condenses onto all interior glass surfaces. The remaining points show BaO/BaCO${}_{3}$ peaks increasing in strength from G1/G2 to G3–G5, and reaching a maximum signal (excepting point G6) at G7, a point on the glass envelope in front of the cathodes. The implication is that the thickness of deposited Ba-containing layers in these regions is no more than 10 nm, as signal from elements present only in the glass substrate is observed. While points G3-G5 and G7 are all 90${}^{\circ }$ away from the flash pans, point G7, in front of the cathodes, is associated with greater Ba signal than points G3-G5, which are located behind the cathodes.

The MgO signal is dramatically enhanced at points G3–G5 and somewhat enhanced at G1–G2. This is likely due to Auger enhancement of the Mg signal due to fluorescence from C-containing layers atop the MgO-containing glass [18]. C-containing layers less than a monolayer thick would yield minimal Auger enhancement of MgO emission (due to reduced intensity of C fluorescence) from the glass, while thicker overlayers (e.g., multiple monolayers) of C-containing material would absorb MgO Auger emissions, also reducing observed signal enhancement. Given that the amount of MgO is likely consistent throughout the glass, variations in MgO peak intensity likely arise from variations in the thickness of C-containing overlayers. While adventitious C is certainly present everywhere, it is likely distributed uniformly on the envelope interior, implying that it is the amount of deposited BaCO${}_{3}$ that varies from region to region. Peak intensity data in Figure 3 for BaO/BaCO${}_{3}$ and (Auger enhanced) MgO indicates that the Ba-containing layer is (i) thin, potentially less than 1 monolayer, at G1 and G2 (limiting Auger enhancement of the MgO signal); (ii) on the order of a monolayer (maximizing enhancement of MgO signal) at G3-G5; and (iii) thicker than a single monolayer—though less than 10 nm thick—at G7 (reducing, but not obstructing, Auger enhancement).

**Figure 4:**
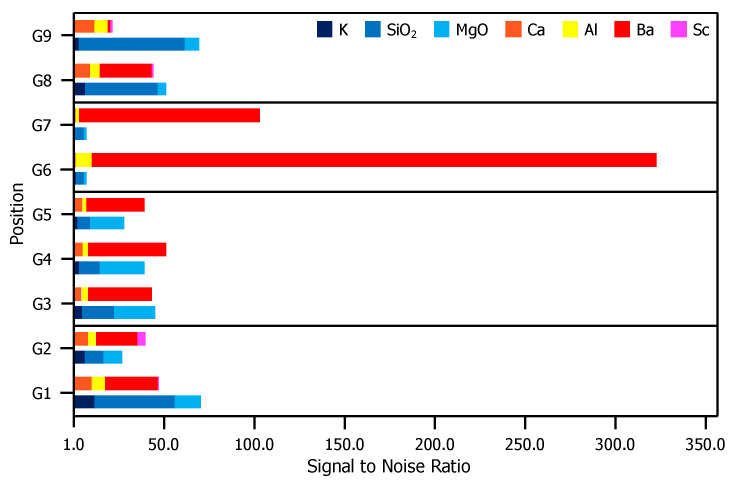
S/N ratios for each relevant peak presented in Figure 3. For elements correlated to multiple peaks, the peak with the greatest height is included. For each position, the top bar is composed of elements found in the impregnate (Ca, Al, Ba, and Sc) and the bottom bar comprises elements found in the glass substrate (K, SiO${}_{2}$, and MgO).

Figure 4 is a comparison of XPS results for the glass envelope. For each peak labeled in Figure 3, the S/N ratio (included in the Appendix A), is plotted for each evaluated position. Contrasts in the S/N ratio observed from position-to-position indicate significantly dissimilar compositions.

### 3.2. Analysis of Anode

**Figure 5:**
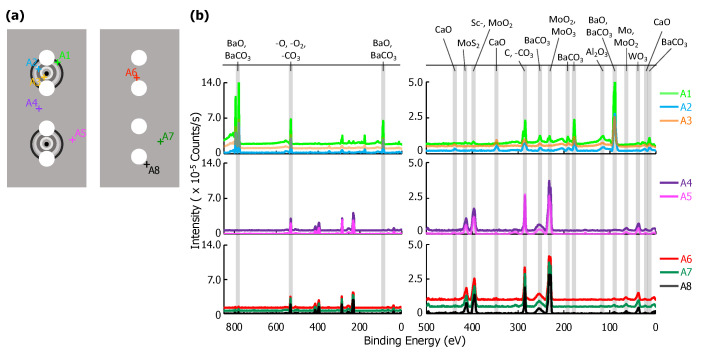
(**a**) A diagram of the anode samples: the left portion of the diagram shows the anode surface facing the cathodes; the right shows the outward-facing surface. Each examined point is plotted, A1–A8. (**b**) On the left, the full XPS survey of each point scan with the three highest-intensity peaks identified. An arbitrary vertical shift is applied to A1, A3, A4, A6, and A7 to improve readability. On the right, a plot of peaks corresponding to binding energies from 500–0 eV. Labels for each relevant peak are included.

XPS results for eight point scans on the anode—five on the cathode-facing side, A1–A5, and three on the opposite side, A6–A8—are shown in Figure 5. The location of each point scan is indicated in the diagram included as Figure 5a, and the two columns of spectra in Figure 5b are as described above for the glass envelope. Arbitrary vertical shifts were applied to improve readability. The eight point scans can be separated into three groups: A1, A2, and A3 are all located on discolored rings discernible on the cathode-facing anode surface (Figure 2). A4 and A5 are also on the cathode-facing side of the anode, but away from discolored rings. A6, A7, and A8 are distributed across the back of the anode, which directly face the glass envelope. Peak binding energies, background-deducted counts, S/N ratios, spectral lines, and species identification for each position can be found in tables located in the Appendix A.

Species related to five elements were observed on the anode: Al, Ba, Ca, Mo, and W. Mo, the primary component of the anode, was observed at all points except A2 and A3. This indicates that deposits in these regions are >10 nm thick and correspond to the discolored rings. Ca signal was observed for points A2 and A3, but were extremely weak or absent at all other points. Al is present only as Al${}_{2}$O${}_{3}$, and only at A1–A3 (in the discolored rings). As Ca is not present at A1, the presence of Al could suggest that the Al and Ca do not originate from the same sources, or that the materials were deposited at different times. Ba-containing compounds (BaO or BaCO${}_{3}$) are detected everywhere, but are particularly strong at points A1–A3. W was also detected, but only at points A4–A8 that are either on the back of the anode, or well away from the deposition rings facing the cathodes.

Sc and Sc${}_{2}$O${}_{3}$ binding energies are similar to those of Mo (within 2 eV of each other at ~400 eV). Signal corresponding to energies of ~400 eV only appear far away from the cathode (A4–A5), or on the back of the anode (A6–A8). At these locations signals from other impregnate elements are absent or trace. In contrast, at locations where other impregnate elements yield strong signals, the signal at ~400 eV is absent. As Sc desorbed from the cathode would be expected to be present on the side of anode facing the cathode and strongest near the cathode or where other impregnate materials is found, we conclude that observed signals ~400 eV arise from Mo.

**Figure 6:**
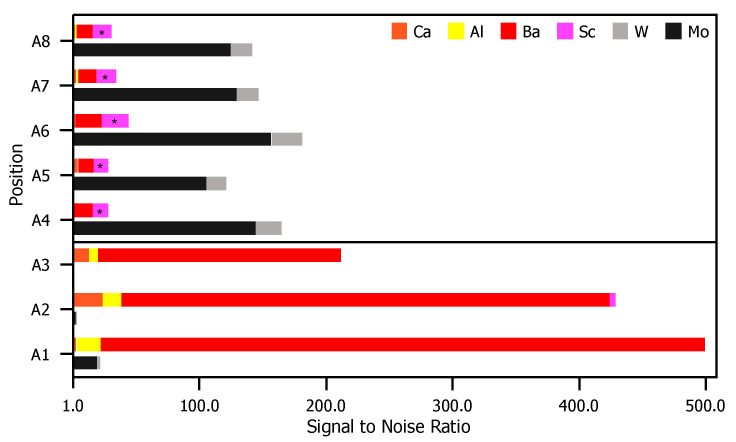
S/N ratios for each relevant peak in Figure 5. For elements correlated to multiple peaks, the peak with the greatest height is included. For each position, the top bar is composed of elements found in the impregnate (Ca, Al, Ba, and Sc) and the bottom bar comprises elements found in the anode substrate (Mg and W).

Figure 6 is a comparison of XPS results for the anode. For each peak labeled in Figure 5, the S/N ratio (included in the Appendix A) is plotted for each evaluated position. Similar to the case for the envelope, there are again contrasts in the observed signal position-to-position. Positions near the pinhole directly opposite the cathodes (A1–A3) shown signal from elements originating in the impregnate, while signal in the other positions (A4–A8) are correlated to elements that are primary components of the substrate.

**Figure 7:**
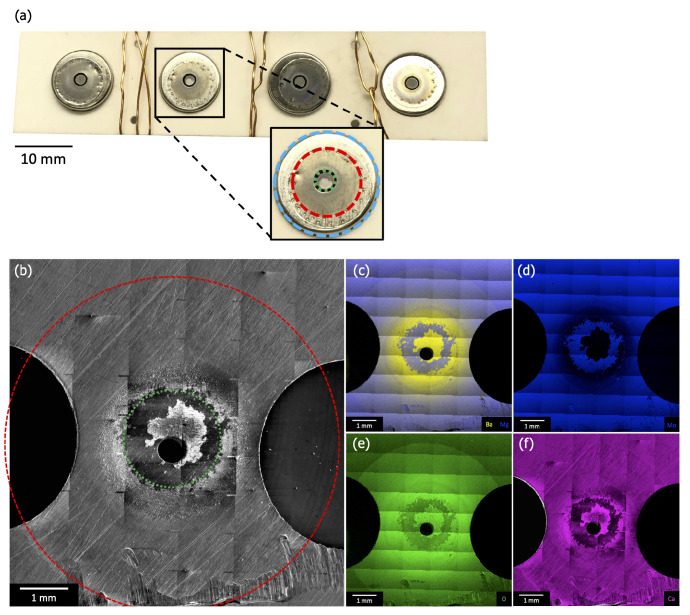
(**a**) A photograph of the cathode assembly before removal of the cathodes but after removal of the anode. This image shows the ceramic spacer penetrated by the cathode housings (blue ring in “zoom-in”) and the cathodes visible through holes in the housing (green ring in “zoom-in”, ~2 mm in diameter). (**b**) Plan-view SEM and (**c**–**f**) EDS maps of Ba/Mg, Mg, O, and Ca from one of the discolored regions of the anode that were observed directly opposite a cathode’s emitting surface (Figure 2). Each image is comprised of multiple scans combined to produce a large area image, allowing for examination of the entire deposition region (red ring in (**b**) and “zoom-in”, ~6 mm in diameter).

Figure 7a is a photograph of the cathode assembly before removal of the cathodes but after removal of the anode plate. The cathode housings (blue ring in “zoom in” image) are visible through holes in the (off-white) ceramic spacer, and the cathodes themselves are visible through holes in this housing (green ring in “zoom-in”, ~2 mm in diameter). The copper wiring is *not* a part of the original cathode assembly, and was added to secure the remaining components after disassembly. Figure 7b–f shows plan-view SEM and EDS results from one of the ring regions of the anode directly opposite the cathodes’ emitting surface (Figure 2). Each image is comprised of multiple scans combined to produce a single large area image, allowing for examination of the entire deposition region (red ring in Figure 7b, ~6 mm in diameter).

SEM and EDS reveal a radial pattern of deposited material consistent with an emission source located under an anode pinhole (diameter ~0.5 mm), and therefore being consistent with the position of the cathode. EDS shows that the deposited material contains Ba, O, and Ca. Large area scans indicate that, beyond the primary deposition area centered on the pinhole (green ring in panel Figure 7b), the deposited layer is thinner than the 1–2 micron sampling depth for EDS. Little or no deposition is observed beyond ~3 mm from the pinhole. This outer deposition region is consistent with features of the cathode housing, as highlighted by equivalent red ring in Figure 7a,b.

The thickness of deposited material increases closer to the primary deposition area (green ring), eventually becoming thick enough to obstruct Mo signal. This area corresponds directly to the size, shape, and position of the cathode itself, as highlighted by equivalent ~2 mm diameter green ring in Figure 7a,b. Portions of the primary deposition area are bare of deposit, as highlighted in Figure 7b–f. Ba, Ca, and Mo scans show that the Mo substrate is exposed in this area, consistent with close examination of SEM images, which show surface scratches on the Mo anode disappearing under the deposited layer, then reappearing. This is hypothesized to be evidence that deposited material flaked off the anode, either during disassembly of the test vehicle or, possibly, during activation or operation.

### 3.3. Glass and Anode Cross Sections

**Figure 8:**
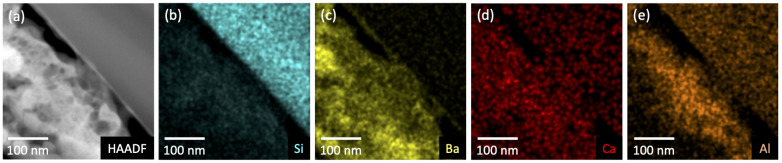
Compositional analysis of the deposit/substrate interface at G6 on the glass envelope. (**a**) High-angle annular dark-field (HAADF) results show a poorly adhered porous layer (bottom left of image) atop a smooth glass substrate (upper right). (**b**–**e**) Elemental mapping shows the distribution of Si, Ba, Ca, and Al within the deposit and substrate.

**Figure 9:**
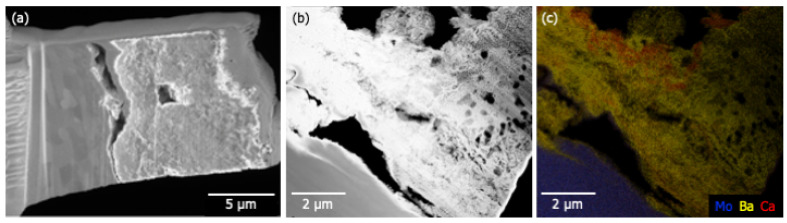
(**a**) SEM results showing the material deposited at point A3 on the anode. The material appears to be poorly adhered to the Mo substrate (left). The total depth of the deposit is between 5 and 8 μm. (**b**) HAADF results indicate a highly porous deposit atop the Mo substrate. (**c**) Elemental mapping shows that Ba is present throughout the deposited material. Thin (~100 nm) Ca-rich layers are observed suggesting that deposition is a multi-stage process.

TEM lift-outs of the anode and glass envelope were extracted using FIB to examine deposit layering. The glass sample was taken from G6, in the Ba-Al flash pan region. The anode sample was taken from A3, close to the pinhole directly above a cathode in the primary deposition area. High-angle annular dark-field (HAADF) images of the glass and anode samples are shown in Figure 8a and Figure 9b, respectively. The substrate—glass or Mo—is visible as the uniform region in the upper right of Figure 8 and lower left in Figure 9b,c, respectively.

Figure 8a shows a poorly adhered porous layer (bottom left) atop a smooth glass substrate (upper right). Elemental mapping of the imaged region are shown in Figure 8b–e; they show Si confined to the glass substrate and Ba to the deposited layer. Ca and Al are present in both materials, with Al enhanced in the near-substrate regions of the deposited layer. Ca is weakly present throughout the glass and the deposit. A similar porous layer ~8 μm thick was also observed on the Mo anode (Figure 9a). Figure 9b shows poor adhesion of the deposited layer and the presence of large voids. These features may have formed during sample preparation, but the numerous smaller voids (less than 1 μm) are likely features formed during deposition. Elemental mapping indicates that Ba is present throughout the deposit. Thin (~100 nm) Ca-rich layers are observed suggesting that deposition may have occurred at different times during activation/operation.

### 3.4. Environmental O${}_{2}$ Availability

Before the glass envelope was broken, the flash region appeared metallic and reflective (seen in Figure 1). Once the envelope was fractured and its interior exposed to air, the region rapidly transitioned to white and dull as residual metallic Ba oxidized to form BaO and BaCO${}_{3}$. The presence of metallic Ba in the sealed glass envelope indicates that after the Ba-Al flash, the residual amount of O${}_{2}$ in the system is extremely low, specifically lower than the threshold O${}_{2}$ partial pressure (that is, chemical potential) required to oxidize Ba. This is consistent with previous results [19] suggesting that O${}_{2}$ partial pressure may significantly affect scandate cathode performance.

### 3.5. Ca and Sc Desorption

The XPS signal associated with Ca was observed from almost every region of the glass envelope, although the signal was generally moderate. Ca was also observed in the exterior reference (G9). This implies that in these regions (everywhere except G6) Ca is either present in the top 10 nm of deposited material or that the deposited layer is less than 10 nm thick. The absence of peaks associated with Ca at G6, the Ba-Al flash region, is notable, especially given that elemental mapping of the cross section show that Ca is plausibly present in at least the first few hundred nanometers of the deposit on the glass substrate at G6 (Figure 8). Scanning electron imaging of the entire FIB lift-out showed the deposit layer to be 2.5–3 μm thick. The fact that Ca is not detected in the top 10 nm of material deposited at G6 (based on XPS, see Figure 3), but *is* detected in the first few hundred nanometers of deposit, may suggest that Ca-containing deposits were formed prior to the Ba-Al flash—that is, before both activation and operation, possibly during pre-activation. These initial Ca-containing deposits would have then been covered by the Ba-Al flash residue.

Ca signal is observed at A2 and A3, which are in or near the primary deposition region (the green ring in Figure 7b). Elemental mapping of an anode lift-out sample originating from A3 shows evidence that Ca deposition did not occur uniformly, seemingly exhibiting layers of Ca-rich deposits (Figure 9). Assuming that the impregnate desorbs relatively uniformly during operation, these striations in deposition composition suggest the possibility that Ca desorption is nonuniform in time. As cathode environmental conditions are uniform during operation this may, in turn, again suggest that Ca is primarily desorbed during pre-activation and activation.

The detection of Sc given the XPS and EDS results is plausible, but highly ambiguous. G2 is the only point on either the glass or the anode to exhibit an isolated peak around 400 eV—the binding energy associated with Sc 2p3/2. On the anode, Sc signal is observed at this energy; however, it is only present at A4–A8, which are away from the cathodes or on the opposite side of the anode. It is more likely that this signal originates from Mo, which is the primary component of the anode. If Sc was desorbing from the cathode, it would be observed in A1–A3. As it is not, the immediate implication is that, unlike Ba, Ca, and Al, either Sc/Sc${}_{2}$O${}_{3}$ desorbs so slowly that even after pre-activation, activation, and ~26,000 h of operation Sc is not detectable, or that Sc does not desorb from the cathode. A dissensus remains in the literature as to the behavior of Sc during operation: some report the presence of Sc at the surface of high-performing scandate cathodes after operation [20], while others hypothesize that Sc readily desorbs based on studies of model Ba-Sc-O on W surfaces [21].

### 3.6. Implications of Anode Deposits

Given the spatial distribution of Ba-containing material deposited on the anode, a crude estimate of the mass deposited on the anode can be made. Approximating the deposited volume as a uniform, 100% dense, 5 μm thick layer of pure BaO (the densest species potentially present, ρ = 5.72 g/cm${}^{3}$) within the primary deposition region (2 mm diameter) yields an estimate of ~0.05 mg of deposited mass. This is likely an overestimate of the amount of deposited material as it ignores porosity in the deposited layer, assumes the deposited material is the most dense of likely species, and neglects potential thinning of the deposited layer towards the edges of primary deposition region. This estimate also assumes that the bare ring evident in Figure 7 was coated, and the deposited material in this region flaked off during post-operation handling. In addition, it should also be noted that it is not clear *when*, during cathode activation and/or operation, deposits on the anode were formed. Despite these limitations, the present calculation is at least a reasonable order-of-magnitude estimate of the mass deposited on the anode. For comparison, similarly manufactured scandate cathodes contain a total of ~4 mg of impregnate prior to activation, as determined by the manufacturer. This suggests that on the order of 1 wt.% of the impregnate is desorbed and deposited on the anode over operating times on the order of 10,000 h.

In addition, EDS analysis strongly indicates that the deposited material primarily consists of Ba/Al/Ca oxide. The presence of an oxide deposit on the anode directly across from the cathode could have significant implications for the apparent emission performance of the cathode. Specifically, a non-conducting, potentially time-dependent coating on the anode will modify details of the applied electric field used to collect emitted current. As noted above, the time and compositional evolution of deposited layers on the anode could not be directly assessed in the present study, but may warrant further examination.

## 4. Conclusions

Species desorbed from a thermionic cathode in a vacuum device must either remain vapor or condense onto exposed surfaces. SEM, EDS, and XPS analyses of surfaces inside a glass test vehicle with enclosed scandate cathodes during pre-activation, activation, and ~26,000 h of operation show that impregnate material was desorbed from the cathodes. This material is observed in small amounts (layers less than 10 nm thick) on all interior surfaces of the enclosing glass envelope. Impregnate is also observed to be deposited on the metal anode in a radial pattern with a maximum thickness on the order of 5 μm. The total amount of impregnate deposited on the anode is estimated to represent on the order of 1% of its original mass.

The thick Ba-Al layer resulting from flash gettering prior to cathode activation was metallic prior to opening the glass test vehicle, and rapidly and visibly oxidized after exposure to air. This demonstrates that the oxygen content in the test vehicle during activation and operation was well below the threshold required to oxidize Ba. In addition, cross-sectional EDS analysis of these layers suggests that Ca desorption is nonuniform during processing and operation of scandate cathodes.

There was little evidence of Sc present on glass envelope and no evidence of its presence on the anode to suggest that any Sc condensed out of the vapor in the glass envelope after pre-activation, activation, and long-term operation is less than 0.1 at.% of any deposited material. That the mole fraction of Sc present in the cathodes after manufacture was at least an order of magnitude higher than this detection threshold indicates that Sc either does not desorb from cathode surfaces, or does so at an undetectable rate.

In sum, these results paint a picture of conditions during scandate cathode operation characterized by very low O${}_{2}$ partial pressure, relatively slow desorption of Ba-, Al-, and Ca-containing impregnate components (on the order of 1 wt.% of initially present material over pre-activation, activation, and operation), minimal desorption of Sc-containing species, and significant coverage of the anode with non-conducting materials. These conditions are complicated by apparent nonuniformities in Ca desorption. Additional studies assessing the condition of the surfaces studied here between activation and operation could yield valuable insight into the different processes occurring at the cathode surface during its lifetime. 

## Appendix A

**Table A1:** XPS data for point G1. Binding energy and Counts taken from the overview spectra. S/N ratios were computed manually. A “-” indicates an S/N ratio below 1.5, implying signal indistinguishable from background. Spectral line and ID were taken from the NIST XPS Database [22]. See text for details.

G1					
**Binding Energy (eV)**	**Counts**	**S/N Ratio**	**Spectral Line**	**ID**	**CAS Registry No.**
779.8	97,480	29	Ba 3d5/2	BaO, BaCO${}_{3}$	1304-28-5, 513-77-9
530.4	849,951	272	O 1s	-O, -CO${}_{3}$	multiple
401.3	8303	-	Sc 2p3/2	Sc${}_{2}$O${}_{3}$	12060081
379.2	13,167	2	K 2s	K-	7440-09-7
351.1	13,095	2	Ca 2p1/2	CaCO${}_{3}$	1304-28-5, 513-77-9
306.8	51,408	14	Mg KLL	Mg-	Ref. [ThermoScientific:TSX:a]
293.8	42,831	12	K 1s	K-	7440-09-7
288.4	45,729	13	C 1s	-CO${}_{3}$	multiple
284.6	145,898	45	C 1s	C (Adv.)	multiple
251.4	8633	-	Ba 4s	Ba-	1304-28-5, 513-77-9
177.7	31,311	8	Ba 4p3/2	Ba-	1304-28-5, 513-77-9
153.8	123,998	38	Si 2s	SiO${}_{2}$	7631-86-9
119.0	29,490	7	Al 2s	Al-	1344-28-1
102.8	144,474	44	Si 2p	SiO${}_{2}$	7631-86-9
89.8	29,723	7	Ba 4d5/2	BaO, BaCO${}_{3}$	1304-28-5, 513-77-9
74.3	18,986	4	Al 2p3/2	Al${}_{2}$O${}_{3}$	1344-28-1
49.9	14,167	2	Mg 2p	Mg-	7439-95-4
28.6	15,608	3	Ba 5s	Ba-	1304-28-5, 513-77-9
24.8	37,001	10	Ca 3p	Ca-	1305-78-8, 471-34-1
13.0	10,717	-	Ba 5p3/2	BaO	1304-28-5

**Table A2:** XPS data for point G2. Binding energy and counts taken from the overview spectra. S/N ratios were computed manually. A “-” indicates an S/N ratio below 1.5, implying signal indistinguishable from background. Spectral line and ID were taken from the NIST XPS Database [22]. See text for details.

G2					
**Binding Energy (eV)**	**Counts**	**S/N Ratio**	**Spectral Line**	**ID**	**CAS Registry No.**
779.8	91,297	23	Ba 3d5/2	BaO, BaCO${}_{3}$	1304-28-5, 513-77-9
530.4	827,893	224	O 1s	-O, -CO${}_{3}$	multiple
401.3	21,795	4	Sc 2p3/2	Sc${}_{2}$O${}_{3}$	12060081
379.2	7056	-	K 2s	K-	7440-09-7
351.1	10,843	-	Ca 2p1/2	CaCO${}_{3}$	1304-28-5, 513-77-9
306.8	44,228	10	Mg KLL	Mg-	Ref. [ThermoScientific:TSX:a]
293.8	29,352	6	K 1s	K-	7440-09-7
288.4	67,720	17	C 1s	-CO${}_{3}$	multiple
284.6	223,906	59	C 1s	C (Adv.)	multiple
251.4	8485	-	Ba 4s	Ba-	1304-28-5, 513-77-9
177.7	31,193	7	Ba 4p3/2	Ba-	1304-28-5, 513-77-9
153.8	127,903	33	Si 2s	SiO${}_{2}$	7631-86-9
119.0	22,730	4	Al 2s	Al-	1344-28-1
102.8	147,314	38	Si 2p	SiO${}_{2}$	7631-86-9
89.8	25,573	5	Ba 4d5/2	BaO, BaCO${}_{3}$	1304-28-5, 513-77-9
74.3	13,905	2	Al 2p3/2	Al${}_{2}$O${}_{3}$	1344-28-1
49.9	12,200	-	Mg 2p	Mg-	7439-95-4
28.6	15,253	2	Ba 5s	Ba-	1304-28-5, 513-77-9
24.8	34,858	8	Ca 3p	Ca-	1305-78-8, 471-34-1
13.0	11,607	-	Ba 5p3/2	BaO	1304-28-5

**Table A3:** XPS data for point G3. Binding energy and counts taken from the overview spectra. S/N ratios were computed manually. A “-” indicates an S/N ratio below 1.5, implying signal indistinguishable from background. Spectral line and ID were taken from the NIST XPS Database [22]. See text for details.

G3					
**Binding Energy (eV)**	**Counts**	**S/N Ratio**	**Spectral Line**	**ID**	**CAS Registry No.**
779.8	240,510	35	Ba 3d5/2	BaO, BaCO${}_{3}$	1304-28-5, 513-77-9
530.4	856,688	130	O 1s	-O, -CO${}_{3}$	multiple
401.3	1422	-	Sc 2p3/2	Sc${}_{2}$O${}_{3}$	12060081
379.2	10,545	-	K 2s	K-	7440-09-7
351.1	37,351	4	Ca 2p1/2	CaCO${}_{3}$	1304-28-5, 513-77-9
306.8	159,155	23	Mg KLL	Mg-	Ref. [ThermoScientific:TSX:a]
293.8	41,495	5	K 1s	K-	7440-09-7
288.4	69,803	9	C 1s	-CO${}_{3}$	multiple
284.6	291,108	43	C 1s	C (Adv.)	multiple
251.4	15,042	-	Ba 4s	Ba-	1304-28-5, 513-77-9
177.7	47,867	6	Ba 4p3/2	Ba-	1304-28-5, 513-77-9
153.8	107,985	15	Si 2s	SiO${}_{2}$	7631-86-9
119.0	33,001	3	Al 2s	Al-	1344-28-1
102.8	125,224	18	Si 2p	SiO${}_{2}$	7631-86-9
89.8	82,798	11	Ba 4d5/2	BaO, BaCO${}_{3}$	1304-28-5, 513-77-9
74.3	22,213	2	Al 2p3/2	Al${}_{2}$O${}_{3}$	1344-28-1
49.9	24,683	2	Mg 2p	Mg-	7439-95-4
28.6	21,599	2	Ba 5s	Ba-	1304-28-5, 513-77-9
24.8	39,232	4	Ca 3p	Ca-	1305-78-8, 471-34-1
13.0	11,826	-	Ba 5p3/2	BaO	1304-28-5

**Table A4:** XPS data for point G4. Binding energy and counts taken from the overview spectra. S/N ratios were computed manually. A “-” indicates an S/N ratio below 1.5, implying signal indistinguishable from background. Spectral line and ID were taken from the NIST XPS Database [22]. See text for details.

G4					
**Binding Energy (eV)**	**Counts**	**S/N Ratio**	**Spectral Line**	**ID**	**CAS Registry No.**
779.8	261,185	43	Ba 3d5/2	BaO, BaCO${}_{3}$	1304-28-5, 513-77-9
530.4	625,698	104	O 1s	-O, -CO${}_{3}$	multiple
401.3	4233	-	Sc 2p3/2	Sc${}_{2}$O${}_{3}$	12060081
379.2	5299	-	K 2s	K-	7440-09-7
351.1	37,681	5	Ca 2p1/2	CaCO${}_{3}$	1304-28-5, 513-77-9
306.8	155,458	25	Mg KLL	Mg-	Ref. [ThermoScientific:TSX:a]
293.8	24,400	3	K 1s	K-	7440-09-7
288.4	57,337	8	C 1s	-CO${}_{3}$	multiple
284.6	224,074	37	C 1s	C (Adv.)	multiple
251.4	14,893	-	Ba 4s	Ba-	1304-28-5, 513-77-9
177.7	44,757	6	Ba 4p3/2	Ba-	1304-28-5, 513-77-9
153.8	61,029	9	Si 2s	SiO${}_{2}$	7631-86-9
119.0	26,412	3	Al 2s	Al-	1344-28-1
102.8	75,559	12	Si 2p	SiO${}_{2}$	7631-86-9
89.8	94,704	15	Ba 4d5/2	BaO, BaCO${}_{3}$	1304-28-5, 513-77-9
74.3	18,264	2	Al 2p3/2	Al${}_{2}$O${}_{3}$	1344-28-1
49.9	28,690	4	Mg 2p	Mg-	7439-95-4
28.6	17,418	2	Ba 5s	Ba-	1304-28-5, 513-77-9
24.8	29,902	4	Ca 3p	Ca-	1305-78-8, 471-34-1
13.0	14,639	-	Ba 5p3/2	BaO	1304-28-5

**Table A5:** XPS data for point G5. Binding energy and counts taken from the overview spectra. S/N ratios were computed manually. A “-” indicates an S/N ratio below 1.5, implying signal indistinguishable from background. Spectral line and ID were taken from the NIST XPS Database [22]. See text for details.

G5					
**Binding Energy (eV)**	**Counts**	**S/N Ratio**	**Spectral Line**	**ID**	**CAS Registry No.**
779.8	210,360	32	Ba 3d5/2	BaO, BaCO${}_{3}$	1304-28-5, 513-77-9
530.4	466,506	72	O 1s	-O, -CO${}_{3}$	multiple
401.3	4487	-	Sc 2p3/2	Sc${}_{2}$O${}_{3}$	12060081
379.2	5591	-	K 2s	K-	7440-09-7
351.1	36,380	5	Ca 2p1/2	CaCO${}_{3}$	1304-28-5, 513-77-9
306.8	127,826	19	Mg KLL	Mg-	Ref. [ThermoScientific:TSX:a]
293.8	19,492	2	K 1s	K-	7440-09-7
288.4	55,283	8	C 1s	-CO${}_{3}$	multiple
284.6	258,716	40	C 1s	C (Adv.)	multiple
251.4	14,716	-	Ba 4s	Ba-	1304-28-5, 513-77-9
177.7	39,347	5	Ba 4p3/2	Ba-	1304-28-5, 513-77-9
153.8	45,632	6	Si 2s	SiO${}_{2}$	7631-86-9
119.0	21,454	2	Al 2s	Al-	1344-28-1
102.8	53,776	7	Si 2p	SiO${}_{2}$	7631-86-9
89.8	84,035	12	Ba 4d5/2	BaO, BaCO${}_{3}$	1304-28-5, 513-77-9
74.3	16,239	-	Al 2p3/2	Al${}_{2}$O${}_{3}$	1344-28-1
49.9	23,692	3	Mg 2p	Mg-	7439-95-4
28.6	14,458	-	Ba 5s	Ba-	1304-28-5, 513-77-9
24.8	22,825	2	Ca 3p	Ca-	1305-78-8, 471-34-1
13.0	13,960	-	Ba 5p3/2	BaO	1304-28-5

**Table A6:** XPS data for point G6. Binding energy and counts taken from the overview spectra. S/N ratios were computed manually. A “-” indicates an S/N ratio below 1.5, implying signal indistinguishable from background. Spectral line and ID were taken from the NIST XPS Database [22]. See text for details.

G6					
**Binding Energy (eV)**	**Counts**	**S/N Ratio**	**Spectral Line**	**ID**	**CAS Registry No.**
779.8	1,610,507	313	Ba 3d5/2	BaO, BaCO${}_{3}$	1304-28-5, 513-77-9
530.4	638,247	123	O 1s	-O, -CO${}_{3}$	multiple
401.3	8617	-	Sc 2p3/2	Sc${}_{2}$O${}_{3}$	12060081
379.2	9488	-0	K 2s	K-	7440-09-7
351.1	11,285	-0	Ca 2p1/2	CaCO${}_{3}$	1304-28-5, 513-77-9
306.8	20,069	2	Mg KLL	Mg-	Ref. [ThermoScientific:TSX:a]
293.8	16,358	-	K 1s	K-	7440-09-7
288.4	112,395	20	C 1s	-CO${}_{3}$	multiple
284.6	73,582	12	C 1s	C (Adv.)	multiple
251.4	60,804	10	Ba 4s	Ba-	1304-28-5, 513-77-9
177.7	181,032	33	Ba 4p3/2	Ba-	1304-28-5, 513-77-9
153.8	15,477	-	Si 2s	SiO${}_{2}$	7631-86-9
119.0	57,889	9	Al 2s	Al-	1344-28-1
102.8	35,347	5	Si 2p	SiO${}_{2}$	7631-86-9
89.8	508,840	97	Ba 4d5/2	BaO, BaCO${}_{3}$	1304-28-5, 513-77-9
74.3	5132	-	Al 2p3/2	Al${}_{2}$O${}_{3}$	1344-28-1
49.9	8596	-	Mg 2p	Mg-	7439-95-4
28.6	26,415	3	Ba 5s	Ba-	1304-28-5, 513-77-9
24.8	19,788	2	Ca 3p	Ca-	1305-78-8, 471-34-1
13.0	54,584	8	Ba 5p3/2	BaO	1304-28-5

**Table A7:** XPS data for point G7. Binding energy and counts taken from the overview spectra. S/N ratios were computed manually. A “-” indicates an S/N ratio below 1.5, implying signal indistinguishable from background. Spectral line and ID were taken from the NIST XPS Database [22]. See text for details.

G7					
**Binding Energy (eV)**	**Counts**	**S/N Ratio**	**Spectral Line**	**ID**	**CAS Registry No.**
779.8	894,149	100	Ba 3d5/2	BaO, BaCO${}_{3}$	1304-28-5, 513-77-9
530.4	588,807	66	O 1s	-O, -CO${}_{3}$	multiple
401.3	3250	-	Sc 2p3/2	Sc${}_{2}$O${}_{3}$	12060081
379.2	3691	-	K 2s	K-	7440-09-7
351.1	13,139	-	Ca 2p1/2	CaCO${}_{3}$	1304-28-5, 513-77-9
306.8	24,582	2	Mg KLL	Mg-	[18]
293.8	12,261	-	K 1s	K-	7440-09-7
288.4	67,774	6	C 1s	-CO${}_{3}$	multiple
284.6	174,219	19	C 1s	C (Adv.)	multiple
251.4	27,105	2	Ba 4s	Ba-	1304-28-5, 513-77-9
177.7	102,380	10	Ba 4p3/2	Ba-	1304-28-5, 513-77-9
153.8	45,233	4	Si 2s	SiO${}_{2}$	7631-86-9
119.0	29,914	2	Al 2s	Al-	1344-28-1
102.8	65,785	6	Si 2p	SiO${}_{2}$	7631-86-9
89.8	254,384	28	Ba 4d5/2	BaO, BaCO${}_{3}$	1304-28-5, 513-77-9
74.3	13,590	-	Al 2p3/2	Al${}_{2}$O${}_{3}$	1344-28-1
49.9	15,538	-	Mg 2p	Mg-	7439-95-4
28.6	18,906	-	Ba 5s	Ba-	1304-28-5, 513-77-9
24.8	27,997	2	Ca 3p	Ca-	1305-78-8, 471-34-1
13.0	31,267	2	Ba 5p3/2	BaO	1304-28-5

**Table A8:** XPS data for point G8. Binding energy and counts taken from the overview spectra. S/N ratios were computed manually. A “-” indicates an S/N ratio below 1.5, implying signal indistinguishable from background. Spectral line and ID were taken from the NIST XPS Database [22]. See text for details.

G8					
**Binding Energy (eV)**	**Counts**	**S/N Ratio**	**Spectral Line**	**ID**	**CAS Registry No.**
779.8	69,591	29	Ba 3d5/2	BaO, BaCO${}_{3}$	1304-28-5, 513-77-9
530.4	564,110	252	O 1s	-O, -CO${}_{3}$	multiple
401.3	7983	-	Sc 2p3/2	Sc${}_{2}$O${}_{3}$	12060081
379.2	7063	-	K 2s	K-	7440-09-7
351.1	5930	-	Ca 2p1/2	CaCO${}_{3}$	1304-28-5, 513-77-9
306.8	15,260	4	Mg KLL	Mg-	Ref. [ThermoScientific:TSX:a]
293.8	18,593	6	K 1s	K-	7440-09-7
288.4	26,542	10	C 1s	-CO${}_{3}$	multiple
284.6	87,381	37	C 1s	C (Adv.)	multiple
251.4	5031	-	Ba 4s	Ba-	1304-28-5, 513-77-9
177.7	20,833	7	Ba 4p3/2	Ba-	1304-28-5, 513-77-9
153.8	94,998	40	Si 2s	SiO${}_{2}$	7631-86-9
119.0	17,314	5	Al 2s	Al-	1344-28-1
102.8	113,444	49	Si 2p	SiO${}_{2}$	7631-86-9
89.8	20,805	7	Ba 4d5/2	BaO, BaCO${}_{3}$	1304-28-5, 513-77-9
74.3	9327	2	Al 2p3/2	Al${}_{2}$O${}_{3}$	1344-28-1
49.9	7379	-	Mg 2p	Mg-	7439-95-4
28.6	16,843	5	Ba 5s	Ba-	1304-28-5, 513-77-9
24.8	25,318	9	Ca 3p	Ca-	1305-78-8, 471-34-1
13.0	7144	-	Ba 5p3/2	BaO	1304-28-5

**Table A9:** XPS data for point G9. Binding energy and counts taken from the overview spectra. S/N ratios were computed manually. A “-” indicates an S/N ratio below 1.5, implying signal indistinguishable from background. Spectral line and ID were taken from the NIST XPS Database [22]. See text for details.

G9					
**Binding Energy (eV)**	**Counts**	**S/N Ratio**	**Spectral Line**	**ID**	**CAS Registry No.**
779.8	8815	2	Ba 3d5/2	BaO, BaCO${}_{3}$	1304-28-5, 513-77-9
530.4	557,050	287	O 1s	-O, -CO${}_{3}$	multiple
401.3	3551	-	Sc 2p3/2	Sc${}_{2}$O${}_{3}$	12060081
379.2	10,942	3	K 2s	K-	7440-09-7
351.1	19,379	4	Ca 2p1/2	CaCO${}_{3}$	1304-28-5, 513-77-9
306.8	19,842	8	Mg KLL	Mg-	Ref. [ThermoScientific:TSX:a]
293.8	28,937	12	K 1s	K-	7440-09-7
288.4	27,402	12	C 1s	-CO${}_{3}$	multiple
284.6	183,564	93	C 1s	C (Adv.)	multiple
251.4	5120	-	Ba 4s	Ba-	1304-28-5, 513-77-9
177.7	20,551	8	Ba 4p3/2	Ba-	1304-28-5, 513-77-9
153.8	100,824	50	Si 2s	SiO${}_{2}$	7631-86-9
119.0	19,531	8	Al 2s	Al-	1344-28-1
102.8	118,811	59	Si 2p	SiO${}_{2}$	7631-86-9
89.8	6215	-	Ba 4d5/2	BaO, BaCO${}_{3}$	1304-28-5, 513-77-9
74.3	9871	2	Al 2p3/2	Al${}_{2}$O${}_{3}$	1344-28-1
49.9	7944	1	Mg 2p	Mg-	7439-95-4
28.6	8793	2	Ba 5s	Ba-	1304-28-5, 513-77-9
24.8	27,283	12	Ca 3p	Ca-	1305-78-8, 471-34-1
13.0	7479	-	Ba 5p3/2	BaO	1304-28-5

**Table A10:** XPS data for point A1. Binding energy and counts taken from the overview spectra. S/N ratios were computed manually. A “-” indicates an S/N ratio below 1.5, implying signal indistinguishable from background. Spectral line and ID were taken from the NIST XPS Database [22]. See text for details.

A1					
**Binding Energy (eV)**	**Counts**	**S/N Ratio**	**Spectral Line**	**ID**	**CAS Registry No.**
779.8	1,331,531	478	Ba 3d5/2	BaO, BaCO${}_{3}$	1304-28-5, 513-77-9
530.4	498,640	176	O 1s	-O, -CO${}_{3}$	multiple
438.0	20,958	2	Ca 2s	Ca-	1305-78-8, 471-34-1
413.5	25,081	2	Mo 3p1/2	MoS${}_{2}$	1317335
401.3	15,240	-	Sc 2p3/2	Sc${}_{2}$O${}_{3}$	12060081
399.5	29,659	5	Mo 3p3/2	MoO${}_{x}$	18868434
344.9	22,964	3	Ca 2p3/2	Ca-	7440-70-2, 471-34-1
288.4	101,072	31	C 1s	-CO${}_{3}$	multiple
284.6	176,840	59	C 1s	C (Adv.)	multiple
251.4	70,249	20	Ba 4s	Ba-	1304-28-5, 513-77-9
232.0	68,106	19	Mo 3d5/2	Mo-	1313-27-5
177.7	181,507	61	Ba 4p3/2	Ba-	1304-28-5, 513-77-9
116.3	69,055	20	Al 2s	Al-	1344-28-1
89.8	469,156	165	Ba 4d5/2	Ba-	1304-28-5, 513-77-9
63.2	10,215	-	Mo 4s	Mo-	7439-98-7
37.9	19,843	2	W 4f5/2	W-	1314-35-8
24.8	22,290	3	Ca 3p	Ca-	1305-78-8, 471-34-1
13.0	53,831	14	Ba 5p3/2	BaO	1304-28-5

**Table A11:** XPS data for point A2. Binding energy and counts taken from the overview spectra. S/N ratios were computed manually. A “-” indicates an S/N ratio below 1.5, implying signal indistinguishable from background. Spectral line and ID were taken from the NIST XPS Database [22]. See text for details.

A2					
**Binding Energy (eV)**	**Counts**	**S/N Ratio**	**Spectral Line**	**ID**	**CAS Registry No.**
779.8	590,068	387	Ba 3d5/2	BaO, BaCO${}_{3}$	1304-28-5, 513-77-9
530.4	283,671	185	O 1s	-O, -CO${}_{3}$	multiple
438.0	14,643	8	Ca 2s	Ca-	1305-78-8, 471-34-1
413.5	2743	-	Mo 3p1/2	MoS${}_{2}$	1317335
401.3	2342	-	Sc 2p3/2	Sc${}_{2}$O${}_{3}$	12060081
399.5	4563	2	Mo 3p3/2	MoO${}_{x}$	18868434
344.9	37,330	23	Ca 2p3/2	Ca-	7440-70-2, 471-34-1
288.4	45,696	29	C 1s	-CO${}_{3}$	multiple
284.6	55,070	35	C 1s	C (Adv.)	multiple
251.4	20,923	13	Ba 4s	Ba-	1304-28-5, 513-77-9
232.0	3786	-	Mo 3d5/2	Mo-	1313-27-5
177.7	71,340	46	Ba 4p3/2	Ba-	1304-28-5, 513-77-9
116.3	25,375	16	Al 2s	Al-	1344-28-1
89.8	257,945	168	Ba 4d5/2	Ba-	1304-28-5, 513-77-9
63.2	474	-	Mo 4s	Mo-	7439-98-7
37.9	2587	-	W 4f5/2	W-	1314-35-8
24.8	12,712	7	Ca 3p	Ca-	1305-78-8, 471-34-1
13.0	22,443	14	Ba 5p3/2	BaO	1304-28-5

**Table A12:** XPS data for point A3. Binding energy and counts taken from the overview spectra. S/N ratios were computed manually. A “-” indicates an S/N ratio below 1.5, implying signal indistinguishable from background. Spectral line and ID were taken from the NIST XPS Database [22]. See text for details.

A3					
**Binding Energy (eV)**	**Counts**	**S/N Ratio**	**Spectral Line**	**ID**	**CAS Registry No.**
779.8	569,221	192	Ba 3d5/2	BaO, BaCO${}_{3}$	1304-28-5, 513-77-9
530.4	270,703	89	O 1s	-O, -CO${}_{3}$	multiple
438.0	21,532	4	Ca 2s	Ca-	1305-78-8, 471-34-1
413.5	7820	-	Mo 3p1/2	Mo${}_{2}$	1317335
401.3	6847	-	Sc 2p3/2	Sc${}_{2}$O${}_{3}$	12060081
399.5	9360	-	Mo 3p3/2	MoO${}_{x}$	18868434
344.9	51,111	14	Ca 2p3/2	Ca-	7440-70-2, 471-34-1
288.4	45,470	12	C 1s	-COS${}_{3}$	multiple
284.6	54,980	15	C 1s	C (Adv.)	multiple
251.4	26,598	6	Ba 4s	Ba-	1304-28-5, 513-77-9
232.0	12,743	-	Mo 3d5/2	Mo-	1313-27-5
177.7	71,175	214	Ba p3/2	Ba-	1304-28-5, 513-77-9
116.3	31,455	7	Al 2s	Al-	1344-28-1
89.8	210,792	69	Ba 4d5/2	Ba-	1304-28-5, 513-77-9
63.2	5708	-	Mo 4s	Mo-	7439-98-7
37.9	8519	-	W 4f5/2	W-	1314-35-8
24.8	18,925	3	Ca 3p	Ca-	1305-78-8, 471-34-1
13.0	22,423	4	Ba 5p3/2	BaO	1304-28-5

**Table A13:** XPS data for point A4. Binding energy and counts taken from the overview spectra. S/N ratios were computed manually. A “-” indicates an S/N ratio below 1.5, implying signal indistinguishable from background. Spectral line and ID were taken from the NIST XPS Database [22]. See text for details.

A4					
**Binding Energy (eV)**	**Counts**	**S/N Ratio**	**Spectral Line**	**ID**	**CAS Registry No.**
779.8	4067	-	Ba 3d5/2	BaO, BaCO${}_{3}$	1304-28-5, 513-77-9
530.4	235,620	98	O 1s	-O, -CO${}_{3}$	multiple
438.0	3638	-	Ca 2s	Ca-	1305-78-8, 471-34-1
413.5	85,674	35	Mo 3p1/2	MoS${}_{2}$	1317335
401.3	31,362	12	Sc 2p3/2	Sc${}_{2}$O${}_{3}$	12060081
399.5	152,950	63	Mo 3p3/2	MoO${}_{x}$	18868434
344.9	595	-	Ca 2p3/2	Ca-	7440-70-2, 471-34-1
288.4	32,506	13	C 1s	-CO${}_{3}$	multiple
284.6	239,737	100	C 1s	C (Adv.)	multiple
251.4	40,140	16	Ba 4s	Ba-	1304-28-5, 513-77-9
232.0	346,772	145	Mo 3d5/2	Mo-	1313-27-5
177.7	3029	-	Ba 4p3/2	Ba-	1304-28-5, 513-77-9
116.3	111	-	Al 2s	Al-	1344-28-1
89.8	6316	2	Ba 4d5/2	Ba-	1304-28-5, 513-77-9
63.2	19,057	7	Mo 4s	Mo-	7439-98-7
37.9	50,703	20	W 4f5/2	W-	1314-35-8
24.8	5345	-	Ca 3p	Ca-	1305-78-8, 471-34-1
13.0	92	-0	Ba 5p3/2	BaO	1304-28-5

**Table A14:** XPS data for point A5. Binding energy and counts taken from the overview spectra. S/N ratios were computed manually. A “-” indicates an S/N ratio below 1.5, implying signal indistinguishable from background. Spectral line and ID were taken from the NIST XPS Database [22]. See text for details.

A5					
**Binding Energy (eV)**	**Counts**	**S/N Ratio**	**Spectral Line**	**ID**	**CAS Registry No.**
779.8	3122	-	Ba 3d5/2	BaO, BaCO${}_{3}$	1304-28-5, 513-77-9
530.4	209,903	83	O 1s	-O, -CO${}_{3}$	multiple
438.0	12,348	4	Ca 2s	Ca-	1305-78-8, 471-34-1
413.5	62,981	24	Mo 3p1/2	MoS${}_{2}$	1317335
401.3	29,293	10.7	Sc 2p3/2	Sc${}_{2}$O${}_{3}$	12060081
399.5	119,630	46.8	Mo 3p3/2	MoO${}_{x}$	18868434
344.9	1667	-	Ca 2p3/2	Ca-	7440-70-2, 471-34-1
288.4	39,144	14.6	C 1s	-CO${}_{3}$	multiple
284.6	276,679	109.6	C 1s	C (Adv.)	multiple
251.4	34,956	11	Ba 4s	Ba-	1304-28-5, 513-77-9
232.0	267,459	106	Mo 3d5/2	Mo-	1313-27-5
177.7	458	-	Ba 4p3/2	Ba-	1304-28-5, 513-77-9
116.3	2450	-	Al 2s	Al-	1344-28-1
89.8	5059	-	Ba 4d5/2	Ba-	1304-28-5, 513-77-9
63.2	16,425	6	Mo 4s	Mo-	7439-98-7
37.9	41,223	16	W 4f5/2	W-	1314-35-8
24.8	9569	3	Ca 3p	Ca-	1305-78-8, 471-34-1
13.0	309	-	Ba 5p3/2	BaO	1304-28-5

**Table A15:** XPS data for point A6. Binding energy and counts taken from the overview spectra. S/N ratios were computed manually. A “-” indicates an S/N ratio below 1.5, implying signal indistinguishable from background. Spectral line and ID were taken from the NIST XPS Database [22]. See text for details.

A6					
**Binding Energy (eV)**	**Counts**	**S/N Ratio**	**Spectral Line**	**ID**	**CAS Registry No.**
779.8	41,470	12	Ba 3d5/2	BaO, BaCO${}_{3}$	1304-28-5, 513-77-9
530.4	297,219	115	O 1s	-O, -CO${}_{3}$	multiple
438.0	104,198	4	Ca 2s	Ca-	1305-78-8, 471-34-1
413.5	173,984	43	Mo 3p1/2	MoS${}_{2}$	1317335
401.3	91,046	21	Sc 2p3/2	Sc${}_{2}$O${}_{3}$	12060081
399.5	215,924	77	Mo 3p3/2	MoO${}_{x}$	18868434
344.9	51,329	2	Ca 2p3/2	Ca-	7440-70-2, 471-34-1
288.4	134,937	16	C 1s	-CO${}_{3}$	multiple
284.6	329,406	117	C 1s	C (Adv.)	multiple
251.4	138,365	21	Ba 4s	Ba-	1304-28-5, 513-77-9
232.0	399,815	157	Mo 3d5/2	Mo-	1313-27-5
177.7	11,172	-	Ba 4p3/2	Ba-	1304-28-5, 513-77-9
116.3	17,742	-	Al 2s	Al-	1344-28-1
89.8	31,468	4	Ba 4d5/2	Ba-	1304-28-5, 513-77-9
63.2	43,282	9	Mo 4s	Mo-	7439-98-7
37.9	72,769	25	W 4f5/2	W-	1314-35-8
24.8	21,560	3	Ca 3p	Ca-	1305-78-8, 471-34-1
13.0	8257	-	Ba 5p3/2	BaO	1304-28-5

**Table A16:** XPS data for point A7. Binding energy and counts taken from the overview spectra. S/N ratios were computed manually. A “-” indicates an S/N ratio below 1.5, implying signal indistinguishable from background. Spectral line and ID were taken from the NIST XPS Database [22]. See text for details.

A7					
**Binding Energy (eV)**	**Counts**	**S/N Ratio**	**Spectral Line**	**ID**	**CAS Registry No.**
779.8	50,222	13	Ba 3d5/2	BaO, BaCO${}_{3}$	1304-28-5, 513-77-9
530.4	323,093	102	O 1s	-O, -CO${}_{3}$	multiple
438.0	91,101	3	Ca 2s	Ca-	1305-78-8, 471-34-1
413.5	173,163	32	Mo 3p1/2	MoS${}_{2}$	1317335
401.3	89,087	15	Sc 2p3/2	Sc${}_{2}$O${}_{3}$	12060081
399.5	211,275	60	Mo 3p3/2	MoO${}_{x}$	18868434
344.9	48,186	-	Ca 2p3/2	Ca-	7440-70-2, 471-34-1
288.4	135,511	16	C 1s	-CO${}_{3}$	multiple
284.6	330,444	96	C 1s	C (Adv.)	multiple
251.4	130,263	14	Ba 4s	Ba-	1304-28-5, 513-77-9
232.0	403,119	130	Mo 3d5/2	Mo-	1313-27-5
177.7	10,465	-	Ba 4p3/2	Ba-	1304-28-5, 513-77-9
116.3	17,168	-	Al 2s	Al-	1344-28-1
89.8	33,990	5	Ba 4d5/2	Ba-	1304-28-5, 513-77-9
63.2	45,771	8	Mo 4s	Mo-	7439-98-7
37.9	67,575	18	W 4f5/2	W-	1314-35-8
24.8	22,300	2	Ca 3p	Ca-	1305-78-8, 471-34-1
13.0	8566	-	Ba 5p3/2	BaO	1304-28-5

**Table A17:** XPS data for point A8. Binding energy and counts taken from the overview spectra. S/N ratios were computed manually. A “-” indicates an S/N ratio below 1.5, implying signal indistinguishable from background. Spectral line and ID were taken from the NIST XPS Database [22]. See text for details.

A8					
**Binding Energy (eV)**	**Counts**	**S/N Ratio**	**Spectral Line**	**ID**	**CAS Registry No.**
779.8	39,563	12	Ba 3d5/2	BaO, BaCO${}_{3}$	1304-28-5, 513-77-9
530.4	245,649	83	O 1s	-O, -CO${}_{3}$	multiple
438.0	89,354	4	Ca 2s	Ca-	1305-78-8, 471-34-1
413.5	153,977	33	Mo 3p1/2	MoS${}_{2}$	1317335
401.3	84,749	15	Sc 2p3/2	Sc${}_{2}$O${}_{3}$	12060081
399.5	189,684	60	Mo 3p3/2	MoO${}_{x}$	18868434
344.9	37,183	-	Ca 2p3/2	Ca-	7440-70-2, 471-34-1
288.4	108,587	10	C 1s	-CO${}_{3}$	multiple
284.6	269,932	83	C 1s	C (Adv.)	multiple
251.4	109,004	13	Ba 4s	Ba-	1304-28-5, 513-77-9
232.0	356,013	124	Mo 3d5/2	Mo-	1313-27-5
177.7	10,427	-	Ba 4p3/2	Ba-	1304-28-5, 513-77-9
116.3	17,800	2	Al 2s	Al-	1344-28-1
89.8	28,374	4	Ba 4d5/2	Ba-	1304-28-5, 513-77-9
63.2	39,289	7	Mo 4s	Mo-	7439-98-7
37.9	61,333	18	W 4f5/2	W-	1314-35-8
24.8	15,286	-	Ca 3p	Ca-	1305-78-8, 471-34-1
13.0	7225	-	Ba 5p3/2	BaO	1304-28-5
